# Antiviral activity of PHA767491 against human herpes simplex virus in vitro and in vivo

**DOI:** 10.1186/s12879-017-2305-0

**Published:** 2017-03-20

**Authors:** Jue Hou, Zili Zhang, Qiang Huang, Jun Yan, Xiaohu Zhang, Xiaoliang Yu, Guihua Tan, Chunfu Zheng, Feng Xu, Sudan He

**Affiliations:** 1 Cyrus Tang Hematology Center, Collaborative Innovation Center of Hematology, Jiangsu Institute of Hematology, Soochow University, Suzhou, China; 2 Jiangsu Key Laboratory of Preventive and Translational Medicine for Geriatric Diseases, Soochow University, Suzhou, China; 3 Department of emergency medicine, First Affiliated Hospital, Soochow University, 1 Shizhi Rd, Suzhou, Jiangsu China; 4 Jiangsu Key Laboratory of Translational Research and Therapy for Neuro-Psycho-Diseases and College of Pharmaceutical Sciences, Soochow University, Suzhou, China; 5 Institutes of Biology and Medical Sciences, Soochow University, Suzhou, China

**Keywords:** HSV, Viral replication, PHA767491, Anti-HSV, Immediate early gene

## Abstract

**Background:**

Herpes simplex virus (HSV) is a common human pathogen that causes a variety of diseases, including oral-labial, genital lesions and life-threatening encephalitis. The antiviral nucleoside analogues such as acyclovir are currently used in anti-HSV therapies; however, clinical overuse of these drugs has led to the emergence of drug-resistant viral strains. Hence, there is an urgent need to develop new anti-HSV agents.

**Methods:**

To identify novel anti-HSV-1 compounds, we screened the LOPAC small scale library of 1280 bioactive compounds to identify inhibitors of HSV-1-induced necroptosis. Further experiments including western blot analysis, Q-PCR analysis and immunohistochemistry were performed to explore the antiviral mechanism of the compounds.

**Results:**

Here, we identified PHA767491 as a new inhibitor of HSV. PHA767491 potently blocked the proliferation of HSV in cells, as well as HSV induced cell death. Further, we found that PHA767491 strongly inhibited HSV infection post viral entry. Moreover, PHA767491 reduced the expression of viral genes required for DNA synthesis including UL30/42 DNA polymerase and UL5/8/52 helicase-primase complex. The essential immediate early (IE) genes such as *ICP4* and *ICP27* are critical for the expression of the early and late genes. Of note, PHA767491 inhibited the expression of all IE genes of both HSV-1 and HSV-2. Importantly, PHA767491 reduced viral titers in the tissues from the mice infected with HSV-1. Consistently, immunohistochemistry analysis showed that PHA767491 dramatically attenuated expression of viral protein gB in the livers.

**Conclusions:**

Taken together, PHA767491 has potent anti-HSV activity by inhibiting viral replication both in vitro and in mouse model. Thus, PHA767491 could be a promising agent for the development of new anti-HSV therapy.

**Electronic supplementary material:**

The online version of this article (doi:10.1186/s12879-017-2305-0) contains supplementary material, which is available to authorized users.

## Background

Herpes simplex virus (HSV) is a group of common human pathogen that causes a variety of clinical manifestations including oral-labial and genital lesions, karatitis and life-threatening encephalitis [1–3]. After primary infection, HSV usually establishes a latent infection in sensory neurons throughout the entire life of the host. This latent infection can be reactivated, resulting in recurrent diseases. HSV is divided into two serotypes, HSV type 1(HSV-1) and type 2(HSV-2), that are the major causes of oral-labial and genital herpes, respectively. In the world, above 80% adults and 20% adults are infected with HSV-1 and HSV-2, respectively. Immunocompromised patients lean to HSV infection and suffer recurrence [4–7].

HSV is enveloped double-strand DNA virus possessing a large genome of around 150k nucleotides. The HSV genome encodes approximately 80 proteins. HSV-1 and HSV-2 share around 80% sequence identity in the protein-coding region. After the HSV virus enters the cytoplasm of the infected cell, the viral genome is released into cell nucleus [8, 9]. Then the linear viral genome transforms to a circular genome and initiates DNA replication at origins of DNA replication [10]. The viral replication is a precisely organized event. Some HSV viral proteins are known to be necessary for viral DNA synthesis, including proteins encoded by *UL5*, *UL8*, *UL29*, *UL30*, *UL42* and *UL52* genes [11–14]. UL9 assists to unwind the DNA strains by binding to the origins of DNA replication. ICP8, encoded by the *UL29* gene, is the major HSV single-strand DNA-binding protein of HSV. UL30 and UL42 are two subunits of DNA polymerase. UL5, UL8 and UL52 constitute helicase-primase complex. HSV genes are expressed in sequential phases termed immediate early (IE), early and late. There are five IE genes: *ICP0*, *ICP4*, *ICP22*, *ICP27* and *ICP47*. Numerous studies have shown that HSV IE genes play important roles in regulating the expression of viral early genes. Most of the early genes are involved in the viral DNA replication. For example, deletion of *ICP4* or *ICP27* significantly impairs the expression of early and late viral genes [15–17]. Therefore, inhibition of these essential IE genes leads to defective viral replication.

A lot of efforts have been focused on the development of anti-HSV therapeutic agents. The antiviral nucleoside analogue acyclovir is the most common drug used for the treatment of HSV infection. Acyclovir can be phosphorylated by viral thymidine kinase and cellular kinases. The product acyclovir triphosphate selectively inhibits viral DNA polymerase to hinder elongation of viral DNA [18]. Penciclovir and foscarnet have a similar mechanism of action to acyclovir and thus are generally used for the treatment of herpesvirus infections [19, 20]. However, there is increasing evidence that these therapies have led to the emergence of drug-resistant mutant strains of HSV [21]. Therefore, it is an urgent need to develop new effective anti-HSV agents.

PHA767491 is reported as an anti-tumor drug, which induce apoptosis in certain type of cancer cell lines [22–25]. In the current study, we identified PHA767491 as a potent inhibitor of HSV-1 and HSV-2. PHA767491 effectively inhibited the proliferation of HSV and viral replication in multiple cells. PHA767491 showed a strong inhibitory effect on the expression of the essential HSV IE genes such as ICP4 and ICP27, therefore leading to suppression of viral replication. Importantly, PHA767491 significantly attenuated HSV-1 replication in mouse model.

## Methods

### Study design

To identify novel anti-HSV-1 compounds, we screened more than 1000 compounds for some antiviral drugs by using the model in which HSV-1 directly induced necrosis of L929. To test the effect of compounds to suppress HSV, plaque forming assay and west blot assay were performed. We further explored the antiviral mechanism of the compounds by using the experiments including Q-PCR analysis, immunofluorescent staining and immunohistochemistry analysis.

### Viruses and reagents

HSV-1 KOS strain was from Dr. Sandra K. Weller. (University of Conecticut Health Center) and GFP-labeled HSV-1 F strain was from Dr. Chunfu Zheng (Soochow University). LOPAC small scale library of 1280 bioactive compounds, LPS and Poly (I:C) were purchased from Sigma Aldrich. Necrostatin-1 was purchased from Alexis Biochemicals. Z-VAD were purchased from WuXi AppTec. The smac mimetic compound were from Dr. Xiaodong Wang (National institute of biological sciences).

### Antibodies

The following antibodies were used: anti-VP16 monoclonal antibody (ab110226; Abcam), anti-gB monoclonal antibody (ab6505; Abcam), anti-β-actin monoclonal antibody (Sigma-Aldrich), anti-P65 polyclonal antibody (F0514; Santa Cruz), secondary antibody (Sigma-Aldrich), anti-phospho-IκB-α monoclonal antibody (9246; Cell Signaling), anti-Phospho-P65 monoclonal antibody (3033p; Cell Signaling), anti-phospho-JNK monoclonal antibody (9251; cell signaling), anti-ICP6 polyclonal antibody was generated in rabbit by immunization with recombinant ICP6 N-terminal polypeptide. Secondary antibody binding to Alexa Fluor 488 was purchased from Life Technologies.

### Antiviral activity assay

L929 Cells were seeded into 96-well plates at the density of 8 × 10^4^. L929 cells were pretreated with compounds (10μM) for 1h and then were infected with HSV-1(MOI = 2) for addition 18h. Cell viability was determined by using Cell Titer-Glo Luminescent cell viability assay kit (Promega) according to the product instructions. Antiviral activities of compounds were calculated as a percentage of viability of control.

### Western blot analysis

Cell pellet was collected by centrifugation at 13000 × g for 1 min and resuspended in lysis buffer (20mM Tris–HCl, PH 7.4, 150mM NaCl, 10% glycerol, 1% Triton X-100, 1mM Na3VO4, 25mM β-glycerol-phosphate, 0.1mM PMSF, a complete protease inhibitor set) (Roche). The re-suspended cell pellet was vortexed for 20s and lysed on ice for 20 min. Cell lysates were centrifuged at 13000 × g for 20 min at 4°C. The supernatants were collected and subjected to western blot analysis. The proteins was detected by using appropriate antibody.

### Cell infected with GFP-labeled virus imaging

Cell were seeded into the 6-well plates at a density of 3 × 10^6^/well and were infected with GFP-labeled HSV-1(MOI = 2) for 8h. The images were obtained with a Leica DMILLED inverted microscope.

### Q-PCR analysis

Total RNA was extracted with TRIzol (Invitrogen) and reverse-transcribed into cDNA according to the procedure of RevertAid First Strand cDNA kit (Thermo). Gene expression was detected by qPCR analysis with SYBR Green PCR Master Mix (Applied Biosystems). The results were analyzed by Applied Biosystems 7500 Fast Real Time PCR System. The sequence of the PCR primers used in Q-PCR analysis were shown in Table 1.

**Table 1:** List of PCR primers used in Q-PCR analysis

HSV-1 ICP0-F	CCTGTCGCCTTACGTGAACA
HSV-1 ICP0-R	CCATGTTTCCCGTCTGGTCC
HSV-1 ICP4-F	CTATATGAGCCCGAGGACGC
HSV-1 ICP4-R	CGTCTGACGGTCTGTCTCTG
HSV-1 ICP6-F	GAGCCCCTTCGTCATGTTCA
HSV-1 ICP6-R	AGTCAAACGTCTGCCTGGAG
HSV-1 ICP22-F	GAAATCTCCGATGCCACCGA
HSV-1 ICP22-R	TCTGGGGTTTCCAGCGTAAC
HSV-1 ICP27-F	CCGAGCCTCTATCGCACTTT
HSV-1 ICP27-R	GTCCCGATAATGGGGTCCTG
HSV-1 GB-F	GGACATCAAGGCGGAGAACA
HSV-1 GB-R	TTCTCCTTGAAGACCACCGC
HSV-1 ICP47-F	TACCGGATTACGGGGACTGT
HSV-1 ICP47-R	ATAAAAGGGGGCGTGAGGAC
HSV-1 UL5-F	GATGACGATCACGTTGCTGC
HSV-1 UL5-R	CCCTCAGGGAGTTTCCGTTC
HSV-1 UL8-F	ATTTTAGTGGCGGGATGCCA
HSV-1 UL8-R	CCGTTAACATCACCACCGGA
HSV-1 UL9-F	GCAGCAGGCGTAGCATTAAC
HSV-1 UL9-R	GGGTTCACCCGAAAACAACG
HSV-1 UL42-F	TGTTCACCACGAGTACCTGC
HSV-1 UL42-R	TTTCCCCGTACACCGTCTTG
HSV-1 UL52-F	CGTCAAACACAACGTGACCC
HSV-1 UL52-R	GCCAAACGCCCCATCATTTT
HSV-2 VP16-F	AATGTGGTTTAGCTCCCGCA
HSV-2 VP16-R	CCAGTTGGCGTGTCTGTTTC
HSV-2 ICP0-F	CGTCTTGTTCACGTAAGGCG
HSV-2 ICP0-R	GAGGAAGTGTGCCAGGAAGA
HSV-2 ICP22-F	GTGCGTCAACCAGCTCTTTC
HSV-2 ICP22-R	CATGAGGTAGCAGTCTCGCA
HSV-2 ICP27-F	CCCTTTCTGCAGTGCTACCT
HSV-2 ICP27-R	CCTTAATGTCCGACAGGCGT
HSV-2 ICP47-F	TGTGTGGGATTTCGACTCGC
HSV-2 ICP47-R	GCGCGGAGATCCATAAAAGG
GAPDH-F	CAAGAAGGTGGTGAAGCAGGC
GAPDH-R	CATACCAGGAAATGAGCTTGAC

### Drug treatment and mice infection

DMSO or PHA767491 were diluted with sterile phosphate-buffered saline (PBS). Eight-week-old RIP3 KO mice were pretreated with DMSO or PHA767491 (25mg/Kg) via i.p. injection for 1h. Then the mice were infected with HSV-1 with 2 × 10^7^ plaque forming units by i.p. injection. Mice were sacrificed 48 h after the HSV-1 injection and the Livers and spleens of mice were excised for virus titer test or immunohistochemistry analysis.

### Determination of viral titers in organs of mice infected with HSV-1

Livers and spleens of mice infected with HSV-1 were ground to homogenates. The homogenates were thawed and frozen three times to fully release the viral particle. Then the homogenates were spin at 600 × g for 5 min. The supernatant were collected and analyzed by plaque forming assay in vero cells.

### Immunohistochemistry analysis

Liver sections from DMSO or PHA767491 pretreated mice were processed by paraffin embedding and stained with anti-gB antibody. To detect the replication of HSV-1, the slices were visualized with DAB (Genetech).

### Immunofluorescent staining

Cells were seeded in culture plates. After the TNF-α treatment, cells were washed with PBS, followed by fixation in 4% paraformaldehyde for 10 min. Then the cells were washed three times with PBS followed by incubation with 0.25% Triton X-100 in PBS for 10 min. The cells were blocked for 30 min with 5%BSA in PBS and stained with primary and secondary antibody successively. Nuclei was stained with DAPI. Images ware captured with a Leica confocal microscope.

### Statistical analysis

Data of antiviral activity are represented as the mean + SD of duplicates. All experiments were repeated at least twice with same results. Significance of difference was evaluated by T tests (Graphpad prism software).

## Results

### Screening of small-molecule inhibitors against HSV-1

**Fig. 1:**
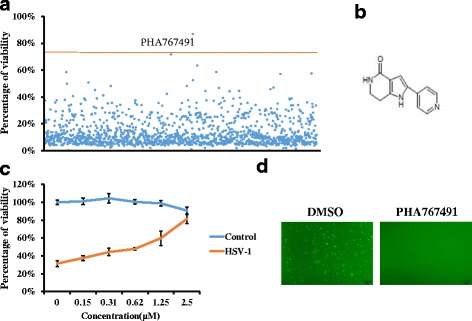
Screening of small-molecule inhibitors against HSV-1 induced necroptosis. **a** L929 cells were pretreated with compounds (10μM) from a library of 1280 chemicals for 1 h and subsequently infected with HSV-1 at a multiplicity of infection (MOI) value of 2 for 20h. Cell viability was assessed by measuring ATP levels. **b** The chemical structure of PHA767491. **c** L929 cells were pretreated with the indicated concentrations of PHA767491 for 1h and then treated with PBS or HSV-1 (MOI = 2) for 20 h and cell viability was measured. **d** L929 cells were pretreated with DMSO or PHA767491 for 1h and then infected with GFP-labeled HSV-1 for 16h. The fluorescence intensity was analyzed via inverted fluorescence microscopy

Our previous study has shown that HSV-1 infection naturally triggers programmed necrosis (termed necroptosis) in mouse fibroblast L929 cells [26]. This process requires effective HSV-1 replication. So compounds with anti-HSV-1 activities can cause reduced necroptosis and increased cell viability of HSV-1-infected L929 cells. To identify novel anti-HSV-1 compounds, we screened the LOPAC small scale library of 1280 bioactive compounds to identify inhibitors of HSV-1-induced necroptosis. PHA767491 was identified as one of the most effective hits that significantly inhibited HSV-1 induced necrosis (Fig. 1a and b). Further, we confirmed that PHA767491 efficiently blocked HSV-1 induced necrosis in a dose-dependent manner, with an estimated IC:50 value of 1.86 μM (Fig. 1c). Next, to evaluate the effect of PHA767491 on HSV-1 proliferation in cells, L929 cells were treated with PHA767491 prior to infection with GFP-labeled HSV-1. As shown in Fig. 1d, PHA767491 potently reduced the proliferation of GFP-labeled HSV-1 in cells, indicating an antiviral activity of PHA767491 against HSV-1.

### PHA767491 does not block TNF-α induced or TLR-induced necrosis

**Fig. 2:**
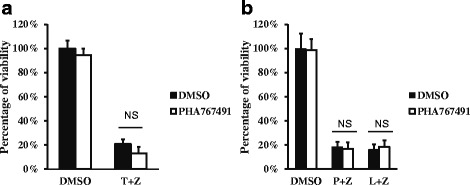
PHA767491 does not block TNF-α induced or TLR-induced necrosis. **a** L929 cells were pretreated with DMSO or PHA767491 (10μM) for 1h and then treated with TNF-α (10ng/ml) plus z-VAD (20μM) for 14 h and cell viability was measured. **b** BMDM were pretreated with DMSO or PHA767491 (10μM) for 1h and then treated with LPS (20ng/ml) or Poly(I:C) (20μg/ml) plus z-VAD(10μM) for 16 h and cell viability was measured. T: TNF-α; P: Poly(I:C); L:LPS; Z: Z-VAD

It is well known that necroptosis can be induced by the activation of death receptors or Toll-like receptors (TLR) [27–31] in addition to HSV-1 infection. We found that PHA767491 did not inhibit TNFR induced necroptosis in L929 cells (Fig. 2a) or TLR-induced necroptosis in macrophages (Fig. 2b), suggesting that PHA767491 does not affect common signaling molecules in the necroptosis pathway. These results suggest that PHA767491 blocks HSV-1 proliferation.

### PHA767491 reduces viral titers and expression of HSV-1 proteins in various cell lines

**Fig. 3:**
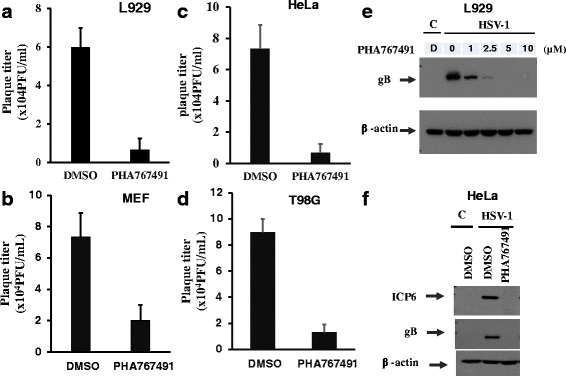
PHA767491 reduces viral titers and expression of HSV-1 proteins in various cell lines. L929 cells and HeLa cells were pretreated with DMSO or PHA767491 for 1h prior to infection with HSV-1(MOI = 2) for 8h. The viral titers for L929 (**a**), MEF (**b**), HeLa (**c**) and T98G (**d**) were analyzed via plaque forming assays. The expression levels of the HSV-1 proteins ICP6 and gB in L929 cells (**e**) or HeLa cells (**f**) were measured by western blot analysis

To further confirm the antiviral activity of PHA767491 against HSV-1, we analyzed viral titer and viral proteins in HSV-1 infected cells in the presence of PHA767491. Using plaques forming assay, we clearly clarified that PHA767491 decreased the viral titer of HSV-1 in mouse L929 and MEFs (Fig. 3a and b). The similar inhibitory effect of PHA767491 on HSV-1 viral titer was observed in human cervical cancer HeLa and gliblastoma T98G cells (Fig. 3c and d). In addition, western blotting analysis showed that PHA767491 efficiently blocked the expression levels of the envelop glycoprotein gB and viral ribonucleotide reductase large subunit ICP6 in all of these examined mouse and human cell lines (Fig. 3e and f). Taken together, these results demonstrate that PHA767491 efficiently reduces HSV-1 viral proteins and viral titers.

### PHA767491 blocks HSV-1 infection post viral entry

**Fig. 4:**
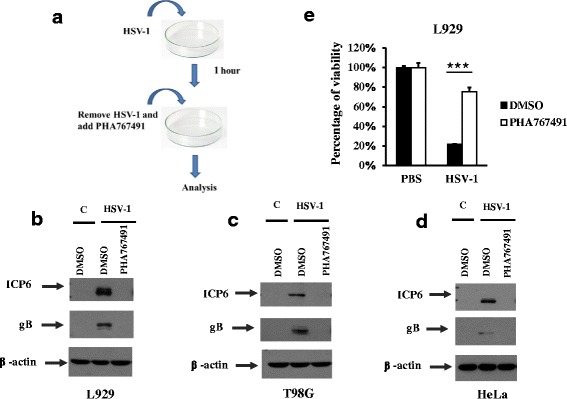
PHA767491 inhibits HSV-1 induced necroptosis and the expression levels of viral proteins after HSV-1 entry. **a** Schematic view. L929 cells (**b**), T98G cells (**c**), and HeLa cells (**d**) were infected with HSV-1(MOI = 4) for 1h. The cells were washed with PBS and then cultured in virus free medium containing with DMSO or PHA767491 for an addition 8h. The expression levels of the HSV-1 proteins gB and ICP6 were measured by western blot analysis. **e** L929 cells were infected with HSV-1 (MOI = 4) for 2h and then washed once with PBS. The cells were then cultured in fresh medium containing DMSO or PHA767491 for an additional 16h. Cell viability was assessed by measuring ATP levels

Next, we investigate whether PHA767491 blocks HSV-1 infection at viral entry or post viral entry. L929 cells were infected with HSV-1 for 1h. After the entry of HSV-1 into the cells, viruses in the medium were washed away and then cultured cells in fresh medium containing DMSO or PHA767491 for additional 8h (Fig. 4a). Notably, PHA767491 efficiently blocked expression of viral proteins such as gB and ICP6 in multiple cell lines after virus entry (Fig. 4b, c and d). Consistently, PHA767491 significantly reduced HSV-1 induced necrosis even after HSV-1 entry (Fig. 4e). Thus, PHA767491 inhibited HSV-1 infection post virus entry.

### PHA767491 inhibits the expression of immediate-early viral genes

**Fig. 5:**
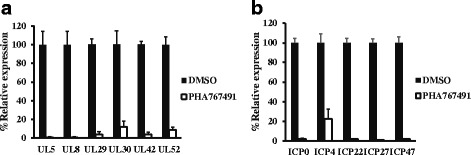
PHA767491 suppresses the expression of HSV immediate early genes. **a**, **b** L929 cells were pretreated with DMSO or PHA767491 for 1h and then infected with HSV-1 (MOI = 2) for 2h. The expression of indicated genes were measured by quantitative PCR

Studies have shown that HSV-1 replication is regulated by some viral proteins such as UL30/42 DNA polymerase and UL5/8/52 helicase-primase complex [11–14]. We evaluated the expression of these genes in the presence of PHA767491. Quantitative PCR analysis showed that PHA767491 remarkably reduced expression of UL5, UL8, UL29, UL30, UL42 and UL52 in the HSV-1 infected cells (Fig. 5a). It is known that HSV immediate early (IE) genes regulate the expression of early and late viral genes. We further examined the effect of PHA767491 on the essential HSV-1 IE genes expression including *ICP0*, *ICP4* and *ICP27*. As shown in Fig. 5b, the expression levels of *ICP0*, *ICP4*, *ICP22*, *ICP27* and *ICP47* were greatly inhibited in cells treated with PHA767491. These results suggest that PHA767491 inhibits HSV-1 replication through the suppression of immediate early gene expression.

### PHA767491 suppresses HSV-2 IE gene expression, viral replication and HSV-2 induced necrosis

**Fig. 6:**
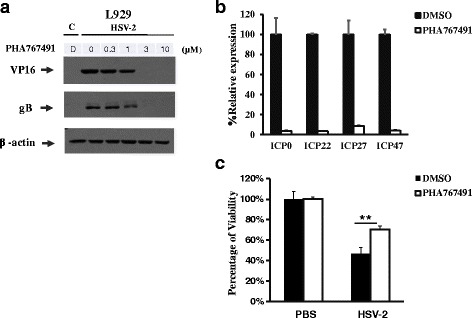
PHA767491 suppresses HSV-2 IE gene expression, viral replication and HSV-2 induced necrosis. **a** L929 cells were pretreated with DMSO or PHA767491 at the indicated concentrations for 1h and infected with HSV-2 (MOI = 2) for 10h, the cell lysates were collected and subjected to western blot analysis. **b** L929 cells were pretreated with DMSO or PHA767491 for 1h and then infected with HSV-2 (MOI = 2) for 2h. The expression of indicated proteins were measured by quantitative PCR. **c** L929 cells were pretreated with DMSO or PHA767491 for 1h and infected with HSV-2 (MOI = 2) for 36h. The cell viability was determined by measuring ATP level

To further address whether PHA767491 could block HSV-2 replication, cells were pretreated with DMSO or PHA767491 and infected with HSV-2. Notably, HSV-2 proteins including VP16 and gB were totally inhibited by PHA767491 at the concentration of 3μM (Fig. 6a). Moreover, we found PHA767491 caused the inhibition of HSV-2 IE gene expression (Fig. 6b). Consistently, PHA767491 significantly blocked HSV-2 induced cell death (Fig. 6c). Thus, PHA767491 is a novel agent against both HSV-1 and HSV-2 viral replication.

### PHA767491 significantly attenuates HSV-1 replication in mouse model

**Fig. 7:**
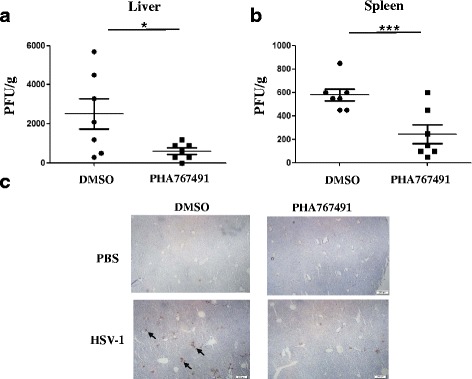
PHA767491 attenuates HSV-1 viral titers in animal model. RIP3 knockout mice were pretreated with DMSO or PHA767491 via intraperitoneal injection and infected with HSV-1 of 2× 107 pfus per mouse by intraperitoneal injection for 2 days. The viral titers in livers (**a**) and spleens (**b**) were determined by plaque assay. **c** The viral titers in the livers from RIP3 knockout mice were measured by immunohistochemistry. Arrows indicated the expression of viral protein gB. The results shown here are representative of seven mice (*n* = 7 per group)

We have previously shown that HSV-1 infection triggers necroptosis in non-natural host mouse cells, but not in natural host human cells [26, 32, 33]. Thus, RIP3-deficient mouse is a more relevant model for the study of HSV-1 infection in vivo. To assess the effect of PHA767491 on HSV-1 replication in mouse model, we injected RIP3-deficient mice with vehicle or PHA767491 followed by HSV-1 infection. Compared with vehicle treatment, PHA767491 reduced viral titer in livers and spleens from RIP3-deficient mice infected with HSV-1 (Fig. 7a and b). Further immunohistochemistry analysis confirmed that PHA767491 dramatically attenuated expression of viral protein gB in the livers (Fig. 7c). These results indicate that PHA767491 exhibits promising therapeutic activity against HSV-1 infection in mouse model.

## Discussion

Uncontrolled HSV infection always lead to some severe diseases such as gingivostomatitis [2, 34, 35] and encephalitis [36–38]. In the current study, we demonstrate PHA767491 as a new anti-HSV agent that potently blocks viral replication of HSV-1 and HSV-2. Our in vivo study revealed a strong inhibition of HSV-1 production by PHA76749 in the mice infection model. Thus, PHA767491 could be a promising agent for the development of new anti-HSV therapy.

One complete cycle for HSV replication need a series of steps including viral attachment and penetration, viral replication, and viral release. Intervention in these processes provides effective protection against HSV infection. Theaflavin digallate has shown to prevent HSV-1 from entering into host to achieve the antiviral activity [39]. Nucleoside analogues such as acyclovir, penciclovir and foscarnet interfere with the viral DNA synthesis to attenuate HSV infections [18–20]. In this study, we found PHA767491 remarkably reduced the expressions of HSV-1 proteins including ICP6 and gB and viral titer in multiple cell lines even after the entry of HSV into the host cells. Similar inhibition of HSV-2 infection by PHA767491 was observed at the post-viral entry stage. These results demonstrate that PHA767491 effectively blocks HSV replication.

HSV replication is regulated by viral proteins. At least several viral proteins are required for viral DNA synthesis, including proteins encoded by *UL5*, *UL8*, *UL29*, *UL30*, *UL42* and *UL52* genes. We found PHA767491 strongly inhibited the expression of these genes. HSV IE genes are expressed earliest after infection without the requirement of viral protein synthesis [3]. The essential IE genes such as *ICP4* and *ICP27* are critical for the efficient expression of the early and late genes. Of note, we found that PHA767491 significantly impaired the expression of all IE genes. The levels of ICP27 RNA and protein were very low or undetectable in the presence of PHA767491. Therefore, PHA767491 disrupts HSV replication by intervening in IE gene expression or upstream signaling molecules regulating IE gene expression.

In addition to viral proteins, NF-κB signals and MAPK signals of host cells are critical for virus replication at the early stage of infection [40–43]. The natural product harmine has been shown to inhibit HSV replication by downregulating both NF-κB and MAPK pathways [44]. However, in our study, PHA767491 did not affect the NF-κB activation (Additional file 1: Figure S1 A and B). Furthermore, we found that PHA767491 had no effects on the activations of ERK, p38 and JNK, three critical kinases of MAPK (Additional file 1: Figure S1 C, D and E). Thus, PHA767491 exhibits anti-HSV activity without affecting cellular NF-κB and MAPK pathways.

## Conclusion

In this study, we identified PHA767491 as a new inhibitor of HSV. PHA767491 potently blocked the proliferation of HSV in various human and mouse cells, including HSV-1 and HSV-2. Moreover, PHA767491 significantly reduced the expression of viral genes required for DNA synthesis including UL30/42 DNA polymerase and UL5/8/52 helicase-primase complex. Moreover, PHA767491 also inhibited the expression of all IE genes of both HSV-1 and HSV-2. Importantly, PHA767491 reduced viral titers in the tissues from RIP3-deficient mice infected with HSV-1. Consistently, immunohistochemistry analysis showed that PHA767491 dramatically attenuated expression of viral protein gB in the livers. Further studies will be required to understand the precise molecular mechanism through which PHA767491 controls the expression of IE genes.

## Additional file

Additional file 1: Figure S1. PHA767491 has no effect on NF-κB and MAPK activation. (DOCX 526 kb)

## Abbreviations

BMDM: Bone marrow derived macrophage
HSV: Herpes simplex virus
LPS: Lipopolysaccharide
MEF: Mouse embryonic fibroblast
Poly (I:C): Poly Inosine acid cytidine acid
RIP3: Receptor-interacting protein kinase 3
TLR: Toll like receptor
TNFR: Tumor necrosis factor receptor
zVAD: Z-val-ala-asp-(o-methlylated)-fluoromethylketone
