# Supplementary material for: Antiviral activity of PHA767491 against human herpes simplex virus in vitro and in vivo
Source: BMC Infect Dis. 2017 Mar 20;17:217. doi: 10.1186/s12879-017-2305-0 (PMC5358049; doi:10.1186/s12879-017-2305-0)
Supplement: Additional file 1: Figure S1. — PHA767491 has no effect on NF-κB and MAPK activation. (DOCX 526 kb) [file 12879_2017_2305_MOESM1_ESM.docx]

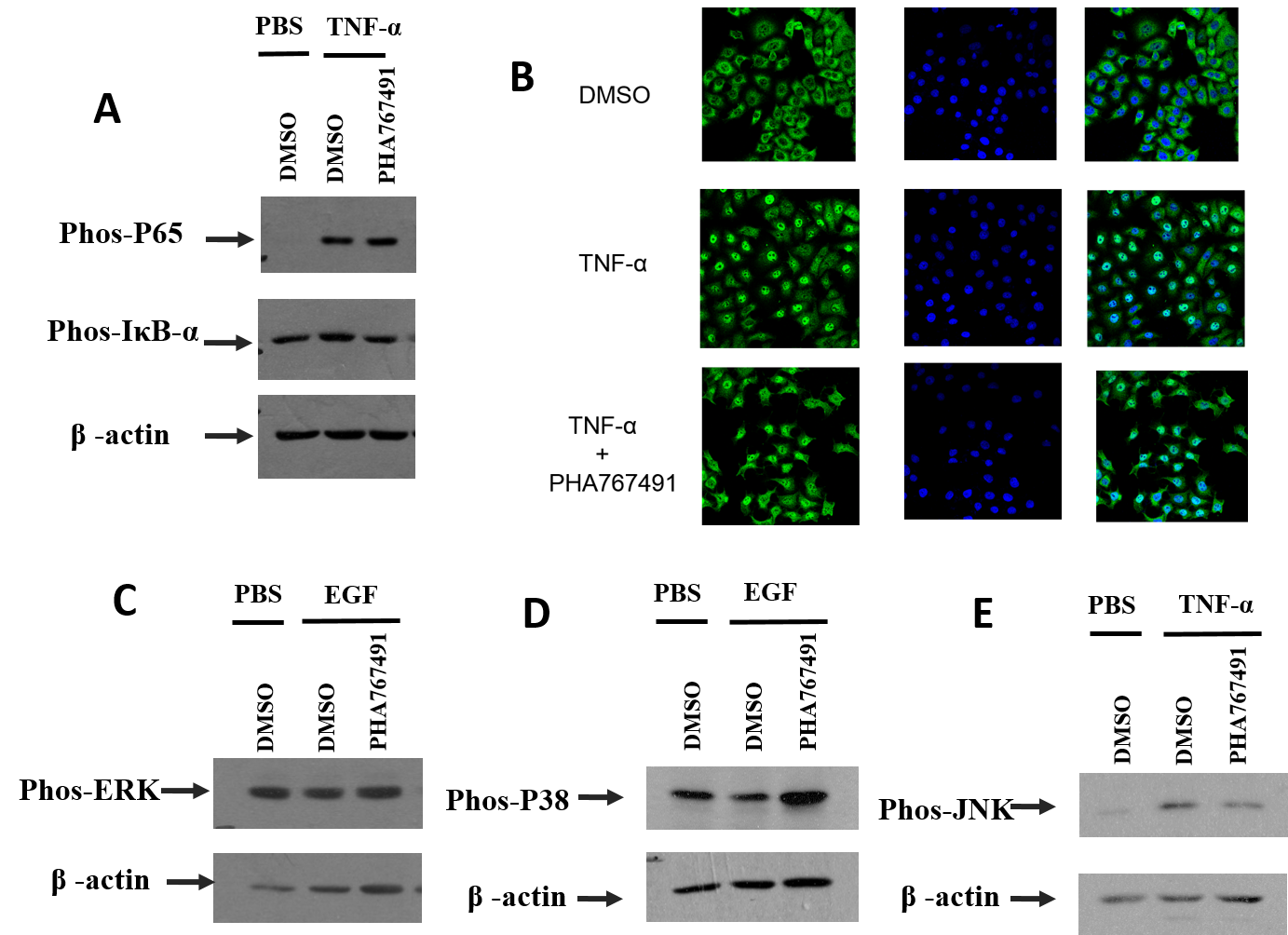


**Figure S1. PHA767491 has no effect on NF-κB and MAPK activation.** MEF cells (A) or HeLa (E) cells were pretreated with DMSO or PHA767491 (10μM) for 1h prior to stimulation with TNF-α (40ng/ml) for 15 minutes. The levels of IκB-α, P65 and JNK phosphorylation were analyzed by western blot analysis. (B) HeLa cells were pretreated with DMSO or PHA767491 (10μM) for 1h prior to stimulation with TNF-α (40ng/ml) for 30 min. Then cell were stained with anti-P65 antibody (green) and DAPI (blue). Then images were analyzed by using fluorescence microscopy. (C, D) RD cells were cultured in serum free medium for 5h. Then cells were treated with indicated compounds and cultured in serum free medium with EGF for addition 3h. The levels of phosphorylated ERK and P38 were analyzed by western-blot analysis.
